# Psychiatric rehabilitation: An innovative program of integrative neurocognitive remediation therapy for patients with cognitive dysfunction

**DOI:** 10.1192/j.eurpsy.2021.417

**Published:** 2021-08-13

**Authors:** A. Rogiers, Z. Van Lieshout, S. Van Eycken, D. Kyndt, J.-C. Le Febvre, A. Lapyte, C. De Gols, C. Kornreich

**Affiliations:** Psychiatry, CHU Brugmann, Brussels, Belgium

**Keywords:** Rehabilitation, Neurocognitive function, daily function, remediation therapy

## Abstract

**Introduction:**

Neurocognitive dysfunction is associated with important socio-professional consequences and diminished quality of life. In the Neurocognitive Remediation Clinic of the Centre Hospitalier Universitaire Brugmann (Brussels, Belgium), patients suffering from neurocognitive dysfunctions related to common mental disorders (e.g. psychotic, mood, adjustment disorders) are considered for Neurocognitive Remediation Therapy (NCRT), combining personalized computerized cognitive training and strategy training, with group sessions of physical rehabilitation and cognitive behavior therapy.

**Objectives:**

This cross-sectional study aims to assess the efficacy of a 12 week (1day/week) NCRT program organized within the day clinic.

**Methods:**

Patients who completed the NCRT between March 2018 and June 2019 were eligible to participate. Efficacy was assessed using the cognitive failure questionnaire (CFQ) and a 17-item questionnaire assessing daily functioning. Current scores on the CFQ were compared to the scores before and after NCRT. Additionally NCF was retrospectively assessed through the neuropsychological test results before and after NCRT.

**Results:**

Of the eligible 38 patients, 27 consented to participate (18 women/9 men); median age was 52 years, range (29-61); median time since stop NCRT was 7 months, range (4-17). Twenty patients (80%) reported improvement in daily function. Subjective neurocognitive function improved significantly immediately after NCRT (t=2.681, df=23, p=0.013) and remained stable at time of assessment (t=2.775, df=24, p=0.011). After NCRT at least 1 neuropsychological subtest normalized in 25 patients (96.15%). Divided attention, long-term visual memory and planning improved in respectively 80%, 75% and 75% of the patients.

**Conclusions:**

Our innovative integrative program improves neuropsychological performances and sustainably ameliorate subjective neurocognitive and daily function.

**Disclosure:**

No significant relationships.

